# Characterization and functional analysis of gerbera* plant defensin* (*PDF*) genes reveal the role of *GhPDF2.4* in defense against the root rot pathogen *Phytophthora cryptogea*

**DOI:** 10.1007/s42994-024-00146-8

**Published:** 2024-03-31

**Authors:** Chunzhen Cheng, Huan Wu, Yongyan Zhang

**Affiliations:** 1 Shanxi Key Laboratory of Germplasm Resources Innovation and Utilization of Vegetables and Flowers, College of Horticulture, Shanxi Agricultural University, Jinzhong, 030801 China; 2 College of Horticulture, Fujian Agriculture and Forestry University, Fuzhou, 350002 China

**Keywords:** Plant defensin, Root rot disease, Antifungal activity

## Abstract

Gerbera (*Gerbera hybrida*), a major fresh cut flower crop, is very susceptible to root rot disease. Although plant defensins (PDFs), a major group of plant antimicrobial peptides, display broad-spectrum antifungal and antibacterial activities, *PDF* genes in gerbera have not been systematically characterized. Here, we identified and cloned nine *PDF* genes from gerbera and divided them into two classes based on phylogenetic analysis. Most Class I *GhPDF* genes were highly expressed in petioles, whereas all Class II *GhPDF* genes were highly expressed in roots. *Phytophthora cryptogea* inoculation strongly upregulated all Class II *GhPDF* genes in roots and upregulated all Class I *GhPDF* genes in petioles. Transient overexpression of *GhPDF1.5* and *GhPDF2.4* inhibited *P. cryptogea* infection in tobacco (*Nicotiana benthamiana*) leaves. Transient overexpression of *GhPDF2.4*, but not *GhPDF1.5*, significantly upregulated *ACO* and *LOX* gene expression in tobacco leaves, indicating that overexpressing *GhPDF2.4* activated the jasmonic acid/ethylene defense pathway and that the two types of GhPDFs have different modes of action. Prokaryotically expressed recombinant GhPDF2.4 inhibited mycelial growth and delayed the hyphal swelling of *P. cryptogea*, in vitro, indicating that GhPDF2.4 is a morphogenetic defensin. Moreover, the addition of GhPDF2.4 to plant culture medium alleviated the root rot symptoms of in vitro*-*grown gerbera seedlings and greatly reduced pathogen titer in *P. cryptogea*-inoculated gerbera roots in the early stages of treatment. Our study provides a basis for the use of GhPDFs, especially GhPDF2.4, for controlling root rot disease in gerbera.

**Supplementary Information:**

The online version contains supplementary material available at 10.1007/s42994-024-00146-8.

## Introduction

Plants produce many low molecular weight peptides for protection against microbial pathogen infection. These peptides include thionine, defensins, hevein-like proteins, and knottin-containing peptides (Nawrot et al. 2014). The activities of these antimicrobial peptides (AMPs) against pathogenic bacteria, fungi, viruses, and parasites have been repeatedly demonstrated (Seo et al. 2012), representing important components of the plant defense system (Emamifar et al. 2021). Plant defensins (PDFs or DEFs), a major group of plant AMPs, display broad-spectrum antimicrobial activity, in vitro (Jha et al. 2010; Dracatos et al. 2016; Kaewklom et al. 2018). PDFs can be divided into two major classes based on their sequences (Zhao et al. 2021). Class I PDFs contain endoplasmic reticulum signal sequences and positively charged defensin domains consisting of ~ 45–55 amino acid residues, whereas Class II PDFs contain an additional C-terminal pro-peptide (CTPP) structure that plays essential roles in intracellular trafficking and detoxification (Dracatos et al. 2013; Lay et al. 2014). Although PDFs from various plant species show limited conservation, they possess similar tertiary structures comprising triple-stranded, antiparallel β-sheets with an α-helix in parallel (CSαβ) that is stabilized by disulfide bridges formed by approximately eight conserved cysteine residues (Sathoff et al. 2019).

The antifungal and antibacterial activities of PDFs and their inhibitory activities against proteinase and insect amylase have been repeatedly confirmed experimentally (de Paula et al. 2008; Mulla et al. 2021). PDFs exert their antimicrobial activities by increasing the permeability of the cell membrane, accelerating the production of reactive oxygen species, and binding to specific receptors (Sher et al. 2019; Mulla et al. 2021). Their antifungal abilities are largely attributed to electrostatic interactions, which prompt rapid initiation of K^+^ efflux and Ca^2+^ uptake to prevent pathogen growth (Ishaq et al. 2019).

Pathogens can infect the cells of the host plant through stomata, where PDFs abundantly accumulate, thereby playing a key role as a defensive barrier to prevent pathogen invasion (Broekaert et al. 1995). A *Medicago truncatula* defensin was shown to inhibit the growth of *Xanthomonas campestris* (Velivelli et al. 2018). Heterologous overexpression of *MtDEF4.2* enhanced the leaf rust disease resistance of transgenic wheat (*Triticum aestivum*) plants (Kaur et al. 2017). Soybean (*Glycine max*) plants overexpressing the *Nicotiana megalosiphon* gene *NmDef02* showed improved resistance against *Phakopsora pachyrhizi* in the field (Soto et al. 2020). Transgenic tobacco plants overexpressing a pepper defensin gene (*J1-1*) and a *Zea mays defensin* gene showed enhanced resistance to *Phytophthora parasitica* (Lee et al. 2018) and *Phytophthora nicotianae* (Al et al. 2020), respectively. The γ-core motifs in MtDef4 and MtDef5 inhibited the growth of *Pseudomonas syringae* (Sathoff et al. 2019). Moreover, chemically synthesized defensins showed broad-spectrum inhibitory activities against pathogenic microorganisms (Kraszewska et al. 2016).

Gerbera (*Gerbera hybrida*), one of the top five fresh cut flowers, worldwide, is used as a model plant for exploring flower development and pigmentation (Wu et al. 2022). However, gerbera plants often suffer from bacterial and fungal diseases, which has seriously restricted the development of the gerbera industry. Among these diseases, root rot disease caused by *Phytophthora cryptogea* and/or *Fusarium oxysporum* is one of the most destructive (Munir et al. 2019). Given the known antifungal properties of PDFs in various plant species, it is likely that PDFs also function in gerbera–pathogen interactions. However, to date, the systematic characterization and functional analysis of the gerbera *PDF* gene family has not been reported.

In this study, based on transcriptome data, we identified and cloned nine gerbera *PDF* genes from *G. hybrida* cv. ‘Linglong’ and investigated their expression patterns in leaves, petioles, and roots of *P. cryptogea*-inoculated and non-inoculated gerbera. To examine their antifungal activities, we performed transient tobacco leaf overexpression-based *P. cryptogea* resistance assays of *GhPDF1.5* and *GhPDF2.4* and in vitro antifungal activity assays of prokaryotically expressed recombinant GhPDF2.4. Our study provides a basis for the future application of GhPDFs in controlling root rot disease and in the breeding of gerbera for increased disease resistance.

## Results

### Identification and cloning of gerbera *PDF* genes

**Fig. 1:**
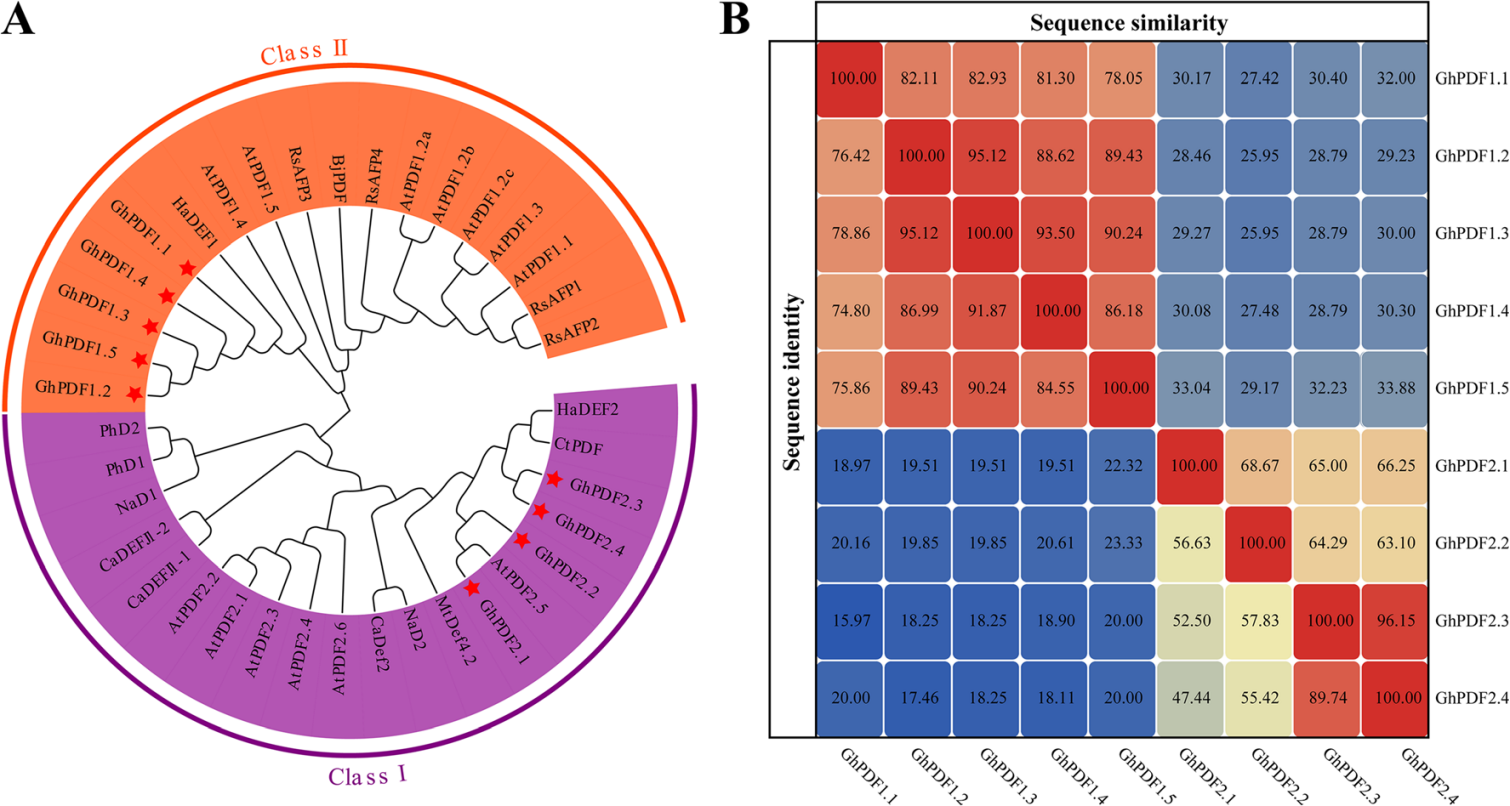
Phylogenetic analysis of PDFs from gerbera and other plant species (**A**) and sequence similarity and sequence identity of GhPDFs (**B**). At: *Arabidopsis thaliana*; Rs: *Raphanus sativus*; Ph: *Petunia hybrida*; Ct: *Carthamus tinctorius*; Ha: *Helianthus annuus*; Zm: *Zea mays*; Bj: *Brassica juncea*; Mt: *Medicago truncatula*; Na: *Nicotiana alata*

**Fig. 2:**
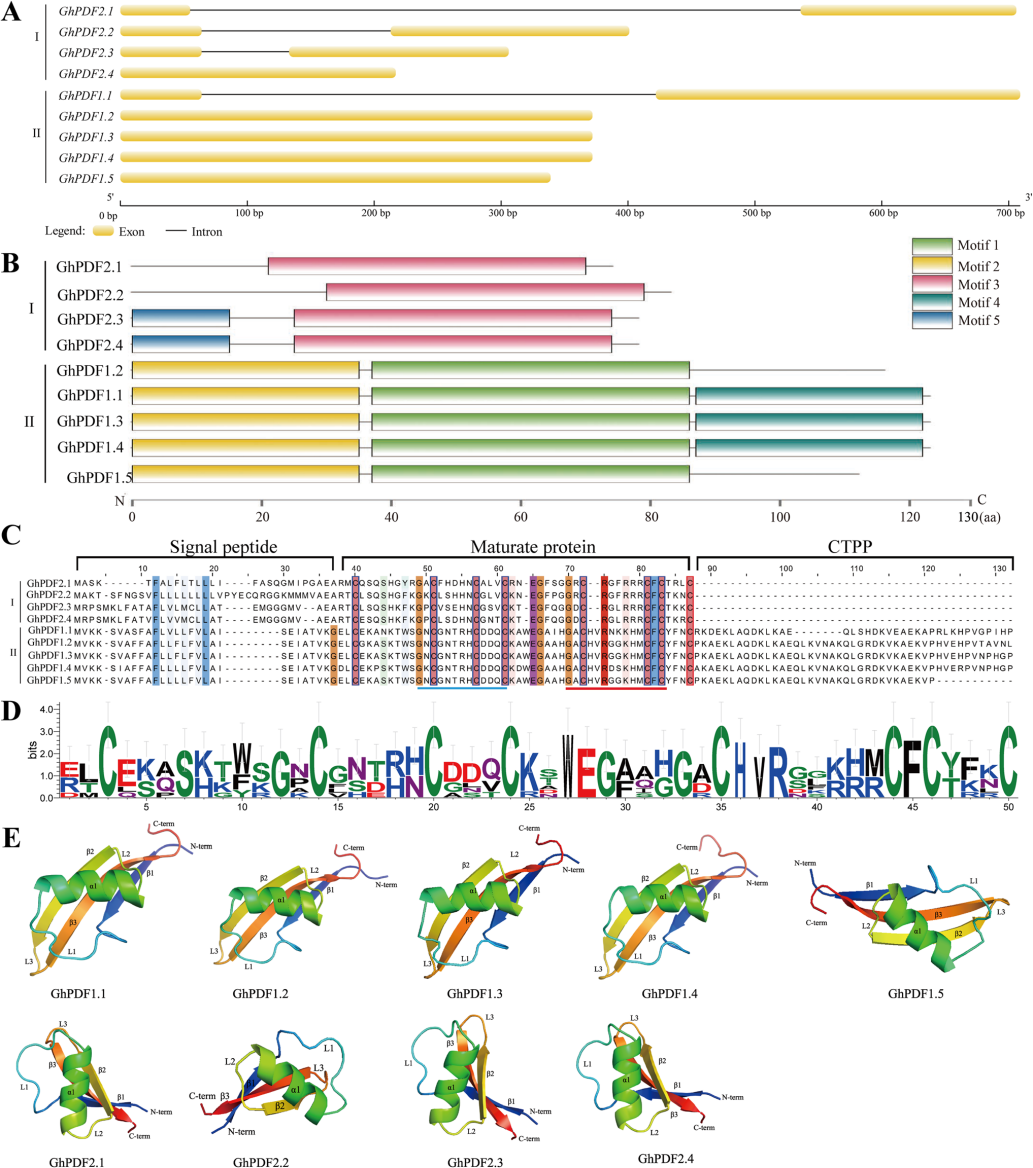
Bioinformatics analysis of *GhPDF* genes and their encoded proteins. **A** Gene structures of *GhPDF* genes. **B** Conserved motifs in GhPDFs. **C** Multiple sequence alignment of GhPDFs. The red line represents the γ core motif sequences, the blue line represents the α core motif sequences. **D** Logos for the mature peptides of GhPDFs. **E** Predicted tertiary structures of the nine GhPDFs

In total, nine gerbera *PDF* genes were identified from our transcriptome data. Using ‘Linglong’ gerbera gDNA and cDNA as templates, we successfully cloned all nine *PDF* genes (Supplemental Fig. S1). We named these genes *GhPDF1.1–1.5* and *GhPDF2.1–2.4* based on the results of phylogenetic analysis and the names of their homologs in *Arabidopsis* (*Arabidopsis thaliana*). The coding sequences of the *GhPDF* genes ranged from 225 to 372 bp long. The gDNA of *GhPDF1.1*, *GhPDF2.1*, *GhPD2.2*, and *GhPD2.3* contained a 69-bp, 481-bp, 149-bp, and 358-bp ‘GT-AG’ type intron, respectively, whereas the other five *GhPDF* genes lacked introns (Figs. 1, 2A).

All GhPDFs were found to contain signal peptides. Notably, the signal peptide sequences of all Class II GhPDFs were the same. All GhPDFs except GhPDF1.1 contained transmembrane structures. All GhPDFs except GhPDF2.1 and GhPDF2.2 were predicted to be hydrophilic proteins, and all GhPDFs were predicted to localize to the extracellular space. Based on phylogenetic analysis, the GhPDFs were categorized into two classes (Fig. 1A). GhPDF2.1 and GhPDF2.2 share the closest relationship with *A. thaliana* AtPDF2.5, while GhPDF2.3 and GhPDF2.4 share the closest relationship with *Carthamus tinctorius* CtPDF. Moreover, GhPDF1.1–1.5 share the closest relationship with *Helianthus annuus* HaDEF1. Amino acid sequence analysis showed that Class I GhPDFs (GhPDF2.1–2.4) contained signal peptides and were mature peptides, while Class II GhPDFs (GhPDF1.1–1.5) also contained a CTPP. The sequence similarities among Class I GhPDFs ranged from 47.44% to 96.15%, with the highest sequence similarity being between GhPDF2.3 and GhPDF2.4 (96.15%) (Fig. 1B). All class II GhPDFs shared sequence similarities higher than 70%, with the highest similarity being between GhPDF1.2 and GhPDF1.3 (95.12%) (Fig. 1B). Thus, the sequence analysis supported the categorization of GhPDFs into two classes.

### Molecular characterization of GhPDFs

In total, we identified five conserved motifs in GhPDFs (Fig. 2B). All Class I GhPDFs contained motif 3, and GhPDF2.3 and GhPDF2.4 also contained motif 5. All Class II GhPDFs contained motif 1 and motif 2, and GhPDF1.2–GhPDF1.4 also contained motif 4. Eight conserved typical cysteines were found in all GhPDFs. The mature peptide domains of Class I and Class II GhPDFs consisted of 47 and 50 amino acids, respectively. The α core motif sequences of all GhPDFs were GXCX5C. The sequences of the γ core region, a key site for the antifungal activity of PDFs (Lacerda et al. 2014), were G[R/D]CRG[F/L]RRCFC for Class I GhPDFs and GACHVR[G/D][G/S]KHMCFC for Class II GhPDFs (Fig. 2C and D). The CTPP of GhPDF1.1 and GhPDF1.5 was composed of 38 and 34 amino acids, respectively, whereas those of the three other Class II GhPDFs contained 45 amino acids (Fig. 2C). Moreover, the GhPDFs shared similar tertiary structures consisting of three antiparallel β-sheets and a parallel α-helix, and the tertiary structures of GhPDFs from the same class were more similar than those of different classes (Fig. 2E).

### Expression analysis of *GhPDF* genes in leaves, petioles, and roots of *P. cryptogea*-inoculated and non-inoculated gerbera

**Fig. 3:**
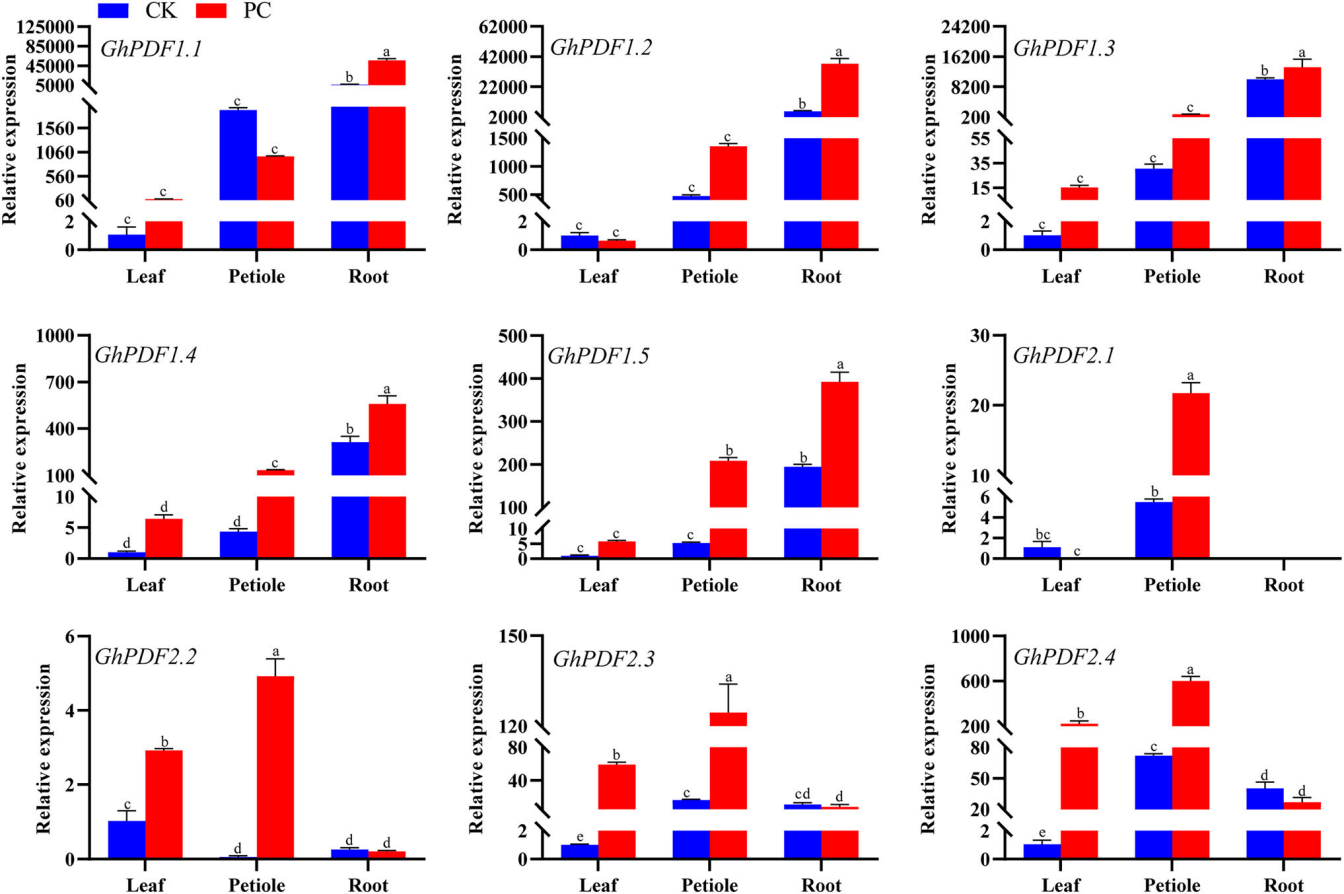
Expression patterns of *GhPDF* genes in leaves, petioles, and roots of *P*. *cryptogea*-inoculated (PC) and non-inoculated healthy control (CK) gerbera. Different letters above the bars indicate a significant difference at the *P* < 0.05 level

Expression analysis of *GhPDF* genes in leaves, petioles, and roots of *P. cryptogea*-inoculated and non-inoculated gerbera revealed that *GhPDF* genes from the two different classes showed different spatial expression patterns and changes in expression in response to *P. cryptogea* infection (Fig. 3).

The expression levels of all Class II *GhPDF* genes were highest in roots and lowest in leaves (Fig. 3). The expression levels of *GhPDF1.1* in the leaves and roots of PC plants were higher than those of CK plants, i.e., 84.35- and 9.28-fold of CK levels, respectively. However, *GhPDF1.1* expression in petioles was downregulated by *P. cryptogea* inoculation to a level of only 50.22% of CK. *GhPDF1.2* expression in petioles and roots was upregulated by 2.87- and 6.43-fold after *P. cryptogea* infection, respectively. However, it was downregulated in the leaves of PC plants to approximately 64% of CK levels. The expression level of *GhPDF1.3* in leaves, petioles, and roots of PC plants was 15.20-, 34.39-, and 1.31-fold that of CK plants, respectively. The expression of *GhPDF1.4* in leaves, petioles, and roots of PC plants was 6.43-, 30.70-, and 1.78-fold that of CK plants, respectively. Finally, the expression level of *GhPDF1.5* in leaves, petioles, and roots of PC plants was 5.79-, 39.77-, and 2.01-fold that of CK plants, respectively.

Except for *GhPFD2.2*, the expression levels of all Class I *GhPDF* genes in CK plants were highest in petioles. *GhPDF2.1* expression was not detected in roots. Following *P. cryptogea* inoculation, *GhPDF2.1* expression in petioles was upregulated to approximately 3.96-fold that of CK plants, but it was downregulated in leaves. The expression levels of *GhPDF2.2* were much lower in petioles and roots than in leaves of CK plants, and its expression in leaves and petioles significantly increased in response to *P. cryptogea* inoculation (*P* < 0.05). Notably, the expression level of *GhPDF2.2* in the petioles of PC plants was 86.73-fold that of CK plants. In PC plants, the expression levels of *GhPDF2.3* in leaves and petioles were 59.08- and 8.10-fold that of CK plants, respectively. *GhPDF2.4* exhibited an expression pattern similar to that of *GhPDF2.3*, i.e., it was significantly upregulated in leaves and petioles of *P. cryptogea*-inoculated gerbera (*P* < 0.05) but did not show significant changes in expression in *P. cryptogea-*infected roots.

### Transient overexpression and functional analysis of *GhPDF1.5* and *GhPDF2.4*

**Fig. 4:**
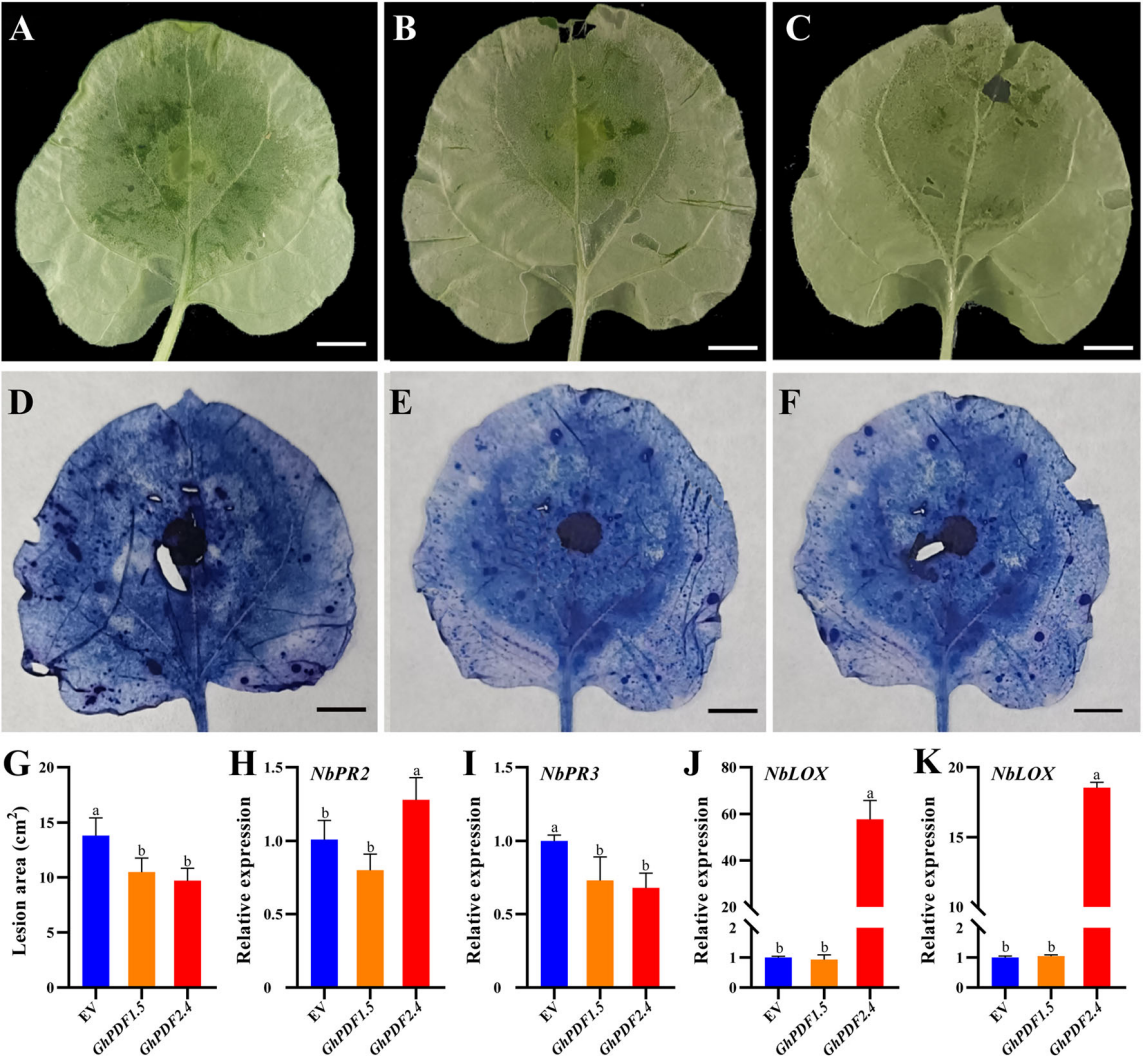
Effects of transient overexpression of *GhPDF1.5* and *GhPDF2.4* on the resistance of tobacco to *P. cryptogea.* **A**–**C** Tobacco leaves overexpressing the empty vector (EV), *GhPDF1.5*, and *GhPDF2.4*, respectively. **D**–**F** Trypan blue staining of tobacco leaves transiently overexpressing EV, *GhPDF1.5*, and *GhPDF2.4*, respectively. Bar, 1 cm. **G** Lesion areas caused by *P. cryptogea* inoculation in the tobacco leaves. **H** Expression of defense-related genes in tobacco leaves after *P. cryptogea* inoculation. Different letters above the columns indicate a significant difference at the *P* < 0.05 level

To verify the functions of *GhPDF* genes, we generated overexpression vectors for a Class I *GhPDF* (*GhPDF2.4*, which is highly expressed in roots and petioles and was upregulated in roots and petioles after *P. cryptogea* infection) and a Class II *GhPDF* (*GhPDF1.5*, which is highly expressed in roots and was upregulated in leaves, petioles, and roots after *P. cryptogea* infection). We transiently overexpressed these vectors in tobacco leaves and subjected them to *P. cryptogea* inoculation (Fig. 4A–F). The lesion areas in tobacco leaves overexpressing *GhPDF1.5* and *GhPDF2.4* were significantly smaller than those in the empty vector (EV) control (*P* < 0.05), i.e., 76.07% and 70.35% of EV values (Fig. 4G), respectively.

We examined the effects of overexpressing *GhPDF1.5* and *GhPDF2.4* on the expression of defense-related genes. No significant changes in the expression levels of *NbLOX*, *NbACO*, or *NbPR2* were detected in tobacco leaves overexpressing *GhPDF1.5*, whereas their expression levels significantly increased in tobacco leaves overexpressing *GhPDF2.4* (*P* < 0.05), accounting for 57.71-, 18.54-, and 1.28-fold of the EV value, respectively (Fig. 4H). Moreover, overexpression of either *GhPDF1.5* or *GhPDF2.4* strongly downregulated the expression of *NbPR3* in tobacco leaves (*P* < 0.05) (Fig. 4H).

### Recombinant GhPDF2.4 protein significantly inhibits the growth of *P. cryptogea* in fungal culture medium

Recombinant GhPDF2.4 production in *E. coli* was successfully induced by 1.0 mM IPTG treatment at 25 °C for 4 h (Supplemental Fig. S2). Based on SDS-PAGE, we confirmed the purified protein to be approximately 23 kDa in size (5.13 kDa GhPDF2.4 + 18.3 kDa Trx-His-S tag) (Supplemental Fig. S3), indicating that we had successfully obtained the prokaryotically expressed recombinant GhPDF2.4 protein.

**Fig. 5:**
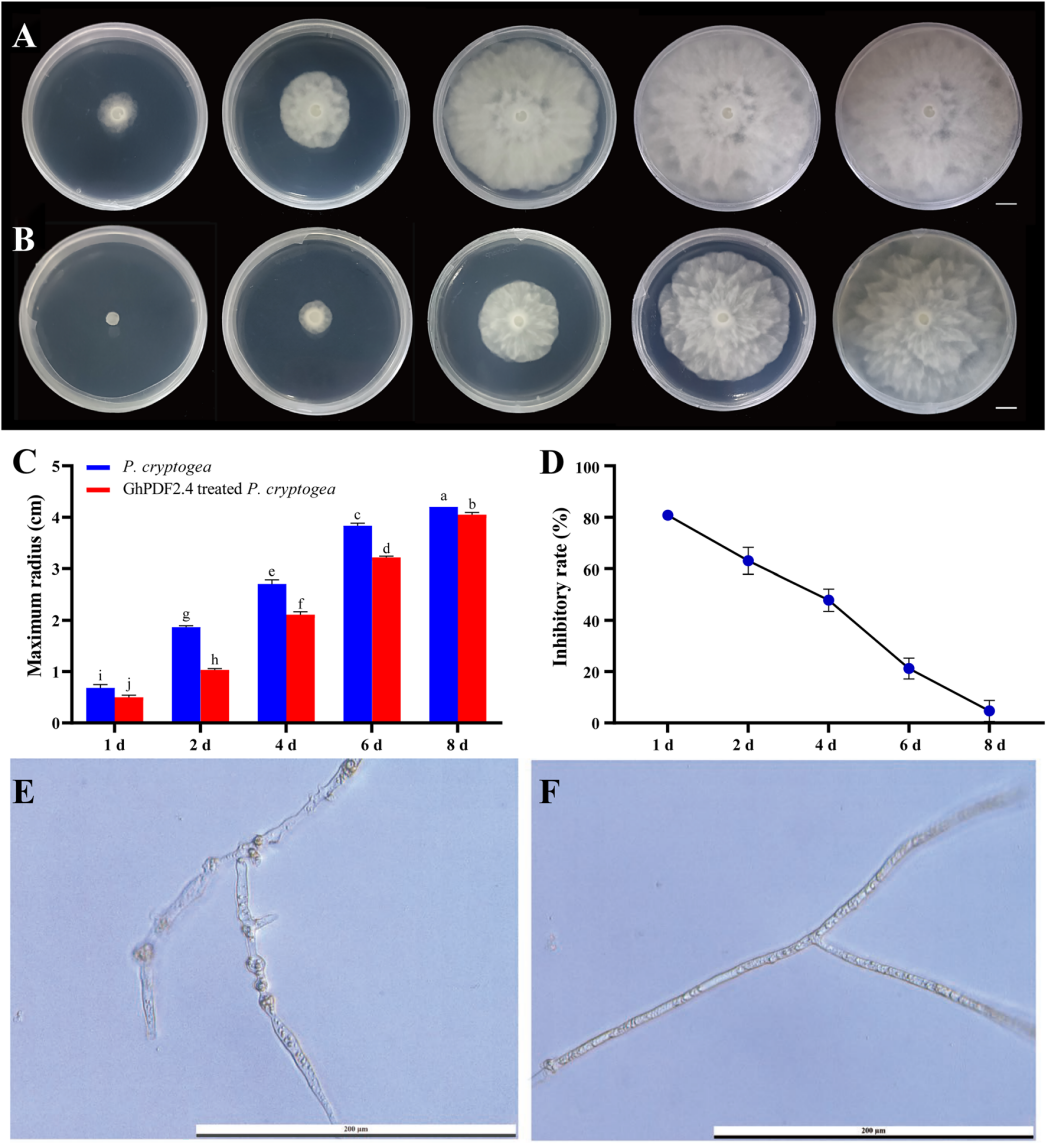
The effect of GhPDF2.4 on the growth of *P. cryptogea.* **A** *P. cryptogea* on PDA medium. **B** GhPDF2.4-treated *P. cryptogea* on PDA medium. The images from left to right were taken at 1, 2, 4, 6 and 8 dpc, respectively. **C** Maximum radius of *P. cryptogea* colonies. Different letters above the columns indicate a significant difference (*P* < 0.05). **D** Rate of inhibition by purified GhPDF2.4 purified protein*.* **E** Normal mycelia of *P. cryptogea* grown on PDA medium for 10 d. F: Mycelia of GhPDF2.4-treated *P. cryptogea*. Bars: A and B, = 1 cm; E and F, = 200 μm

To explore the antifungal activity of GhPDF2.4 in vitro, we performed growth inhibition assays of *P. cryptogea* using 200 μL purified recombinant GhPDF2.4 protein at a concentration of approximately 1 mg·mL^−1^. In the first 2 days post culture (dpc), the colony radius of GhPDF2.4-treated *P. cryptogea* was significantly smaller than the control (Fig. 5A and B), i.e., only 19.19% and 36.93% of the control radius, respectively. At 4 dpc, the colony radius of GhPDF2.4-treated *P. cryptogea* was approximately 52.20% that of the control. At 6 dpc, the *P. cryptogea* colony had covered the entire culture dish. However, the GhPDF2.4-treated *P. cryptogea* colony covered the entire culture dish at 8 dpc. The inhibitory effects of GhPDF2.4 protein weakened over time (Fig. 5C and D), with 80.80% and 63.07% inhibition on the first two days of treatment, declining to 47.79%, 21.18%, and 4.70% at 4, 6, and 8 dpc (Fig. 5D), respectively.

At 10 dpc, we observed the mycelia of GhPDF2.4-treated and control *P. cryptogea* (Fig. 5E and F). We observed hyphal swelling in control *P. cryptogea* mycelia but not in GhPDF2.4-treated *P. cryptogea*, indicating that the protein delayed the hyphal swelling of *P. cryptogea*.

### GhPDF2.4 treatment alleviates the root rot symptoms of in vitro*-*grown gerbera seedlings

**Fig. 6:**
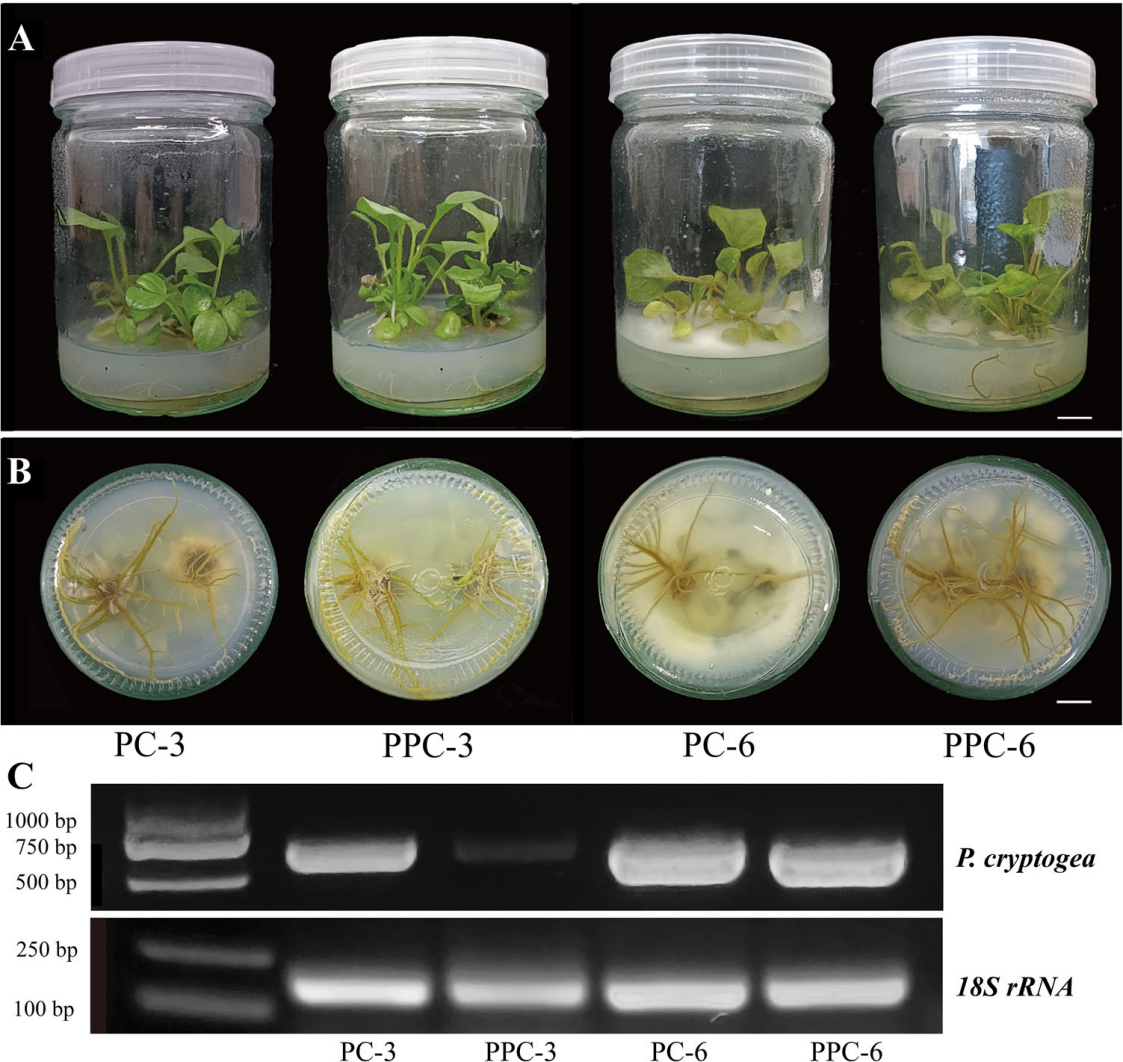
Effects of GhPDF2.4 on root rot resistance in in vitro*-*grown gerbera seedlings. **A** *P. cryptogea-*inoculated in vitro*-*grown gerbera seedlings not treated or treated with GhPDF2.4 at 3 and 6 days post inoculation (dpi); **B** Roots of *P. cryptogea*-inoculated in vitro*-*grown gerbera seedlings not treated or treated with GhPDF2.4 at 3 dpi and 6 dpi. Bar, 1 cm. **C** Semi-quantitative PCR to detect *P. cryptogea* titer in gerbera roots

Finally, we investigated the antifungal activity of recombinant GhPDF2.4 in in vitro*-*grown gerbera seedlings by adding purified recombinant protein into the plant culture medium. Notably, its addition significantly alleviated root rot symptoms in in vitro*-*grown gerbera plantlets in the early stages of treatment (Fig. 6A). Mycelia were clearly observed in PC medium at 3 dpi, whereas no mycelia were observed in control PPC medium (Fig. 6B). At 6 days after *P. cryptogea* inoculation, abundant hyphae appeared in the medium in both groups, but there were much fewer hyphae in control PPC medium than in PC medium. At 6 dpi, both PC and PPC plants showed wilted and water-soaked roots, but PPC plants were much more upright than PC plants (Fig. 6B).

We measured the titers of *P. cryptogea* in the roots of PC and PPC plants by semi-quantitative PCR. *P. cryptogea* was detected in all samples, but the *P. cryptogea* titer was significantly lower in PPC than in the PC group, especially at 3 dpi. These results indicate that the addition of GhPDF2.4 protein inhibited *P. cryptogea* infection during the early stages of treatment (Fig. 6C), which is similar to the results of the in vitro antifungal experiment.

## Discussion

PDFs, one of the most important types of plant AMPs, are increasingly being recognized as potential antifungal agents due to their broad-spectrum antifungal activities (Tetorya et al. 2023). PDFs are encoded by multigene families in plants. For example, 15, 12, 37, and 16 *PDF* family members have been identified in the genomes of *Arabidopsis* (Thomma et al. 2002), peanut (Zhao et al. 2021), allotetraploid rapeseed (Liu et al. 2021), and *M. truncatula* (Hanks et al. 2005), respectively. In this study, we successfully identified and cloned nine *PDF* genes from gerbera, which could be divided into two classes.

### The two classes of *GhPDF* genes show varied expression patterns in different organs and in response to *P*. *cryptogea* infection

Gene expression analysis revealed that most Class I *GhPDF* genes were highly expressed in gerbera petioles and all Class II *GhPDF* genes were highly expressed in roots. The Class I gene *GhPDF2.1* showed no expression in roots. Thus, the spatial expression patterns of the two classes of *GhPDF* genes are different. In several other plants, different *PDF* genes also show different spatial expression patterns. For example, oat (*Avena sativa* L.) *AsDef1* is most highly expressed in developing seeds but shows no expression in leaves, stems, or roots (Emamifar et al. 2021), whereas the expression of sunflower *HaDef1* is only detected in roots and mature leaves (de Zelicourt et al. 2007).

*PDF* genes function in many plant responses to both abiotic and biotic stresses. The expression of *M. truncatula* defensin genes was induced by a variety of biotic and abiotic stress treatments (Liu et al. 2021)*.* The expression levels of peanut *AhDef1.5*, *AhDef1.6*, *AhDef2.1*, and *AhDef2.2* in roots, stems, and leaves were significantly upregulated by *R. solanacearum* infection (Zhao et al. 2021). Spruce (*Picea asperata*) *PaDef* was significantly upregulated (by 3.34-fold) after 2 months of *Lophodermium piceae* infection (Liu et al. 2022b). In the current study, we found that after *P. cryptogea* infection in gerbera, most *GhPDF* genes were upregulated in leaves and petioles, suggesting that their upregulation might be attributed to the systemic resistance of gerbera. In the roots, however, only Class II *GhPDF* genes were upregulated by pathogen infection, indicating that the changes in expression of the two classes of *GhPDF* genes in gerbera roots varied in response to *P. cryptogea* infection.

### GhPDFs have great potential for use as bioinspired fungicides to control root rot disease in gerbera

Although the nine GhPDFs shared relative low sequence similarities, their tertiary structures, especially among members from the same class, were quite similar. Like PDFs from other plant species, GhPDFs exhibit a cysteine-stabilized CSαβ structure consisting of one α-helix and three antiparallel β-strands (Kovaleva et al. 2020). All GhPDFs were found to contain eight conserved typical cysteine residues, which can form four disulfide bonds: Cys1-Cys8, Cys2-Cys5, Cys3-Cys6, and Cys4-Cys7 (Ishaq et al. 2019). Positively charged amino acid residues in PDFs are conducive to their interactions with negatively charged microbial membranes (Sagaram et al. 2011; Xu et al. 2023). Moreover, the γ-core motif, a group of positively charged amino acid residues, is common to all disulfide-containing antimicrobial peptides and contributes greatly to their antifungal activity (Spelbrink et al. 2004; de Paula et al. 2011; de Oliveira Mello et al. 2019). Our study revealed that the γ core motifs of Class I GhPDFs contained more positively charged residues than those of Class II GhPDFs. Consistent with this, the antifungal effect of overexpressing *GhPDF2.4* was better than that of *GhPDF1.5* in tobacco leaves, indicating that their different antifungal activities might be related to the abundance of the positively charged amino acid residues in the γ core motif.

*PDF* has been recognized as a marker of the jasmonic acid (JA)/ethylene (ET) defense pathway (Li et al. 2020; Verly et al. 2020). LOXs and ACOs, which are key factors influencing the JA and ET biosynthesis pathway, also play key roles in plant defense responses against biotic and abiotic stress (Houben et al. 2019; Viswanath et al. 2020). Here, the transcription levels of *NbLOX* and *NbACO* significantly increased in tobacco leaves overexpressing *GhPDF2.4*, indicating that its overexpression activated the JA/ET defense pathway. However, no significant changes in expression of *NbLOX* or *NbACO* were detected in tobacco leaves overexpressing *GhPDF1.5*, suggesting that the modes of action of the two *GhPDF* genes differ.

Over the past two decades, PDFs have frequently and successfully been applied in plant disease resistance engineering. Transgenic tobacco plants overexpressing the maize (*Zea mays*) gene *ZmDEF1* showed enhanced tolerance against *P. parasitica* (Wang et al. 2011). Fusarium wilt and Alternaria leaf spot disease resistance greatly improved in transgenic *Colocynthis citrullus* overexpressing the wasabi (*Wasabia japonica*) *defensin* gene (Ntui et al. 2010). Transgenic soybean overexpressing *NmDef02* showed enhanced resistance to Asian soybean rust caused by *Phakopsora pachyrhizi* and anthracnose caused by *Colletotrichum truncatum* (Soto et al. 2020). In addition, transgenic banana overexpressing petunia *PhDef1* and *PhDef2* exhibited strong resistance to Fusarium wilt (Ghag et al. 2012).

In addition to exhibiting broad-spectrum antifungal activity at micromolar concentrations in vitro (Nanni et al. 2013; Kerenga et al. 2019; Tetorya et al. 2023), PDFs are relatively stable small molecules due to their disulfide bridge-stabilized structure and relatively high amounts of cationic charged residues (Soto et al. 2020). These advantages make PDFs quite suitable for direct application in disease control. Prokaryotic expression, an effective strategy for expression of AMPs, has frequently been utilized to produce PDFs (Ceballo et al. 2022). Treatment with OsDEF7 and OsDEF8 significantly inhibited the growth of *Xanthomonas oryzae* and *F. oxysporum*; defensins purified from *Petunia hybrida* and *Nicotiana alata* exhibited strong antifungal activities against *F. oxysporum* and *Botrytis cinerea *in vitro (Lay et al. 2003); recombinant ZmDEF1 inhibited the growth of *P. parasitica* (Wang et al. 2011); and PaDef inhibited the growth of *Pestalotiopsis neolitseae* (Liu et al. 2022b). In the current study, prokaryotically expressed recombinant GhPDF2.4 significantly inhibited the growth of *P. cryptogea* in fungal culture medium, and its exogenous application alleviated root rot symptoms in in vitro*-*grown gerbera seedlings.

### GhPDF2.4 is a morphogenetic plant defensin

PDFs can be divided into morphogenetic and non-morphogenetic plant defensins based on their functional activities and morphogenic effects on fungal hyphae (Kovaleva et al. 2020). Morphogenetic PDFs inhibit mycelial growth and decrease mycelial branching, whereas non-morphogenetic PDFs only slow hyphal extension without inducing visible morphological changes (Broekaert et al. 1995; Kovaleva et al. 2020). In this study, treatment with recombinant GhPDF2.4 altered the morphology of hyphae and delayed hyphal swelling of *P. cryptogea* in fungal culture medium, indicating that GhPDF2.4 is a morphogenetic plant defensin.

## Conclusions

In this study, we successfully identified and cloned nine *PDF* genes from gerbera. These GhPDFs could be divided into two classes, which exhibited different sequence properties and expression patterns in different gerbera organs and in response to *P. cryptogea* infection. Transient overexpression of *GhPDF1.5* and *GhPDF2.4* inhibited the penetration of *P. cryptogea* in tobacco leaves, with different modes of action. Prokaryotically expressed recombinant GhPDF2.4 significantly inhibited the growth of *P. cryptogea* in fungal culture medium, and its exogenous application alleviated root rot symptoms and reduced pathogen titers in the roots of in vitro-grown gerbera during the early stages of treatment. Moreover, GhPDF2.4 treatment delayed the hyphal swelling of *P. cryptogea*, confirming that GhPDF2.4 functions as a morphogenetic plant defensin. Our findings shed light on the functions of GhPDFs in gerbera–*P. cryptogea* interactions and provide a basis for the future application of GhPDFs, especially GhPDF2.4, to control gerbera root rot disease.

## Materials and methods

### Plant materials and treatments

The transplanted gerbera ‘Linglong’ (*G. hybrida* cv. ‘Linglong’) seedlings used in this study were provided by the Flower Research Institute, Yunnan Academy of Agricultural Sciences. Seedlings were grown in a greenhouse at 28 °C, 60% to 80% relative humidity, and a photoperiod of 12 h light/12 h dark (1500 ± 200 lx) for two months.

For *P. cryptogea* inoculation, fungal solution (containing 1 × 10^6^ spores/mL) was applied to the soil close to gerbera roots at a final concentration of approximately 100 mL per kilogram soil. Gerbera plants treated with an equal volume of potato dextrose broth (PDB) solution were used as controls. Leaf, petiole, and root samples from *P. cryptogea*-inoculated (PC) and non-inoculated healthy control (CK) gerbera seedlings were collected at 18 days post inoculation (dpi), that is, when the PC plants began to display root rot symptoms. Each sample was independently collected, and all samples were fast-frozen in liquid nitrogen and stored at − 80 °C until use. For each group, three biological replicates were used.

### Identification and cloning of *PDF* genes from gerbera

The 15 *Arabidopsis* PDF family protein sequences were downloaded from TAIR (http://arabidopsis.org/) and used as query sequences to search against the gerbera proteins with e-value ≤ 1 × 10^−5^ as the criterion. The hidden Markov model file for PDF (PF00304) was downloaded from the Pfam database (http://pfam.xfam.org/) and searched against the gerbera protein data using HMMER software (e value ≤ 1 × 10^−5^). The conserved domain database (CDD, https://www.ncbi.nlm.nih.gov/cdd) was used to confirm the presence of the conserved gamma-thionin domain, and sequences without this domain were removed from further analysis.

Total DNA was isolated from gerbera leaves using the CTAB method. An RNAprep Pure Plant Kit (TIANGEN, Beijing, China) was used to isolate total RNA from different gerbera samples. The cDNA used for gene cloning was synthesized using a RevertAid First Strand cDNA Synthesis Kit (Thermo Scientific, Beijing, China). Gene-specific primers used to clone the gDNA and cDNA sequences of *GhPDF* genes were designed using DNAMAN software (Supplemental Table S1). The 25-µL amplification system consisted of 1 µL gDNA or cDNA, 1 µL each forward and reverse primers (10 μM), 12.5 µL 2 × SYBR Green mix, and 9.5 µL ddH_2_O. PCR amplification conditions were set as follows: pre-denaturation at 95 °C for 3 min; 34 cycles of 95 °C for 30 s, 56–60 °C for 1 min, and 72 °C for 1 min; and final extension at 72 °C for 5 min. The amplified products were gel purified using a GeneJET Gel Extraction Kit (Thermo Fisher Scientific, Beijing, China). After TA cloning, positive clones were sent to Qingke Biotechnology (Fuzhou) Co., Ltd. for sequencing verification. The sequences of the *GhPDF* genes were submitted to GenBank under accession numbers OP470820–OP470828.

### Bioinformatic analysis of *GhPDF* genes and their encoded proteins

The basic physicochemical properties, subcellular localizations, signal peptides, and transmembrane structures of the GhPDFs were predicted as described by Zhang et al. (2021). Heatmap embedded in TBtools was used to visualize the sequence similarities and identities among *GhPDF* genes. GSDS 2.0 (http://gsds.gao-lab.org/) was used to draw gene structure diagrams. MEME (https://meme-suite.org/meme/tools/meme) was used to identify conserved motifs, with motif length set at 10–50 amino acid residues. WebLogo 3 (http://weblogo.threeplusone.com/create.cgi) was used to draw logos for the conserved domain of GhPDFs.

PDF protein sequences from *Arabidopsis*, radish, and several other plant species were downloaded from NCBI (https://www.ncbi.nlm.nih.gov/) and subjected to multiple sequence alignment. MEGA-X was used to construct a neighbor-joining (NJ) phylogenetic tree (Poisson model, complete deletion, and bootstrap = 1000). The phylogenetic tree was visualized using Evolview (http://www.evolgenius.info/evolview/).

### Quantitative real time PCR (qRT-PCR)

A TransScript All-in-One First-Strand cDNA Synthesis SuperMix for qPCR (One-Step gDNA Removal) kit (TransGen, Beijing, China) was used to separately synthesize cDNAs from different samples. Gene-specific primers used for qRT-PCR were designed using DNAMAN based on the sequences of the *GhPDF* genes (Supplemental Table S1). Amplification was performed on a LC480 real-time quantitative fluorescent PCR instrument (Roche Diagnostics). The qRT-PCR reaction system consisted of 10 µL SYBR Premix ExTaq fluorescent dye (TaKaRa, Beijing, China), 7.4 µL ddH_2_O, 0.8 µL each of upstream and downstream primers (10 μM), and 1 µL cDNA template. The qRT-PCR conditions were as follows: pre-denaturation at 95 °C for 30 s and then 40 cycles of denaturation at 95 °C for 10 s, annealing at 60 °C for 20 s, and extension at 72 °C for 20 s. Using *18S rRNA* as an internal reference gene, the relative expression levels of *GhPDF* genes in different samples were calculated using the 2^−∆∆Ct^ method (Chen et al. 2011). The results were analyzed using IBM SPSS Statistics version 26.0 (Armonk, NY, USA) and graphed using GraphPad Prism 8.0.2 (San Diego, CA, USA).

### Recombinant vector construction

*GhPDF1.5* and *GhPDF2.4* were individually subcloned using gene-specific primers (Supplemental Table S1), with the TA plasmid carrying the target gene used as a template, digested using *Kpn*I and *Pst*I, purified, and inserted into pCAMBIA1301. Recombinant vectors and the empty vector pCAMBIA1301 (EV) were individually transformed into *Agrobacterium tumefaciens* strain GV3101. Recombinant pET-32a-GhPDF2.4 vectors were constructed for the prokaryotic expression of *GhPDF2.4*.

### *P. cryptogea* resistance assays in tobacco (*Nicotiana benthamiana*) leaves

*A. tumefaciens* carrying EV, pCAMBIA1301-*GhPDF1.5*, and pCAMBIA1301-*GhPDF2.4* were grown on Luria–Bertani (LB) agar plates containing 50 ng/mL kanamycin and 50 ng/mL rifampicin for ~ 36 h. A single colony was picked and cultured at 28 °C at 200 rpm until OD_600_ reached 0.8–1.0. The bacterial solution was centrifuged at 5000 g for 5 min, resuspended, adjusted to OD_600_ = 0.7–1.0 in MES solution (containing 200 mM MES, 100 mM MgCl_2_, and 100 μM acetosyringone, pH = 5.8), and incubated at 28 °C for 2–3 h to allow leaf infiltration.

Tobacco leaves were infiltrated with *Agrobacteria* solution using a needleless syringe and incubated in the dark for 2 d. The leaves were collected, inoculated with *P. cryptogea* by placing a *P. cryptogea* plug (5 mm diameter) on the injected lower epidermis, and placed on a 90 mm diameter petri dish. To maintain a high humidity environment, the leaf petioles were wrapped with moist cotton (Wang et al. 2023). Three days after inoculation, phenotypic observation, trypan blue staining, and gene expression analysis were performed. Lesion area caused by *P. cryptogea* was calculated by converting pixels to inches in Photoshop CS6 (San Jose, CA, USA). Total RNA was extracted from different tobacco samples using the TRIzol method. Primers used for qRT-PCR of defense-related genes (*NbPR2*, *NbPR3*, *NbACO*, and *NbLOX*) and the reference gene (*NbEF-1α*) (Sun et al. 2023) are shown in Supplemental Table S1. All tests were performed in three biological replicates, each comprising a mixed sample of six leaves.

### Prokaryotic expression and purification of GhPDF2.4

The recombinant pET-32a-GhPDF2.4 and empty pET-32a vectors were separately transformed into *Escherichia coli* BL21(DE3) (Cheng et al. 2023). Recombinants were screened on medium containing 100 μg/mL ampicillin, and single colonies were cultured in liquid LB medium and detected by PCR using specific primers for *GhPDF2.4* (Supplemental Table S1). Positive clones were inoculated into LB liquid culture medium (containing 100 μg/mL ampicillin) and cultured with shaking at 200 rpm at 37 °C until the OD_600_ reached 0.6–0.8. Protein expression was induced by adding 1.0 mM isopropyl-beta-D-thiogalactopyranoside (IPTG), followed by further culturing at 25 °C and 200 rpm for 4 h. The bacteria were centrifuged at 10,000 rpm at 4 °C for 15 min, resuspended and ultrasonicated, and purified using nickel affinity (Ni–NTA) resin (Liu et al. 2022a). The concentration of purified protein was measured using an ultra-micro nucleic acid protein detector (Thermo Fisher Scientific, USA). To confirm protein quality, 15% sodium dodecyl sulfate–polyacrylamide gel electrophoresis (SDS-PAGE) and Coomassie Blue (G-250) staining were used.

### Antifungal activity assays of GhPDF2.4

For the antifungal activity assay, 200 μL of purified GhPDF2.4 at a concentration of ~ 1 mg·mL^−1^ was spread onto PDA medium. A 0.5-cm *P. cryptogea* plug was inoculated into the center of the PDA medium. *P. cryptogea* plugs cultured on PDA medium without protein addition were used as controls. The plates were inverted, placed in a biochemical incubator at 28 °C in the dark, and photographed daily. The largest radius of each fungal colony was measured at 1 day, 2 days, 4 days, 6 days, and 8 days post culture (dpc). The rate of inhibition was calculated according to the following formula: Inhibition rate (%) = (radius of control fungal colony—radius of treated fungal colony)/radius of control fungal colony × 100. At 10 dpc, *P. cryptogea* mycelia were collected and observed under an Olympus optical microscope (DMI8, LEICA, Germany). All tests were performed with at least three replicates.

To explore the effect of prokaryotically expressed GhPDF2.4 protein on root rot resistance in gerbera, 200 μL of ~ 1 mg·mL^−1^ recombinant GhPDF2.4 was placed evenly near the roots of in vitro-grown gerbera seedlings before *P. cryptogea* inoculation (PPC); seedlings inoculated with *P. cryptogea* (PC) were used as controls. The growth and disease symptoms of *P. cryptogea*-treated plants were observed daily. For each treatment group, six bottles of in vitro-grown gerbera seedlings were used, each containing at least two seedlings.

At 3 dpi and 6 dpi, the roots of PC and PPC gerbera were collected to measure *P. cryptogea* titer. DNA was extracted from the roots of PC and PPC gerbera by the CTAB method and adjusted to a concentration of 50 ng·μL^−1^. Semi-quantitative PCR was used to detect the titers of *P. cryptogea* in different samples using *P*. *cryptogea ITS* primers (PC1: 5’-CGGCCTGGGCTAGTAGCGTA-3’; PC2: 5’-TCCACCCCAGCTTACGCCAG-3’, target length = 125 bp) (Safaiefarahani et al. 2016) with gerbera *18S rRNA* used as reference gene. The amplification conditions were as follows: 95 °C for 3 min; 34 cycles of 95 °C for 30 s, 55 °C for 1 min, and 72 °C for 1 min; and a final extension at 72 °C for 10 min.

## Supplementary Information

Below is the link to the electronic supplementary material.

Supplementary file1 (PNG 989 KB)

Supplementary file2 (PNG 612 KB)

Supplementary file3 (TIF 1660 KB)

Supplementary file4 (DOCX 35 KB)

## Data Availability

The datasets generated and analyzed during this study are available from the corresponding author on reasonable request.
