# Correction: Characterization and functional analysis of gerbera plant defensin (PDF) genes reveal the role of GhPDF2.4 in defense against the root rot pathogen Phytophthora cryptogea

**DOI:** 10.1007/s42994-024-00179-z

**Published:** 2024-08-26

**Authors:** Chunzhen Cheng, Huan Wu, Yongyan Zhang

**Affiliations:** 1 Shanxi Key Laboratory of Germplasm Resources Innovation and Utilization of Vegetables and Flowers, College of Horticulture, Shanxi Agricultural University, Jinzhong, 030801 China; 2 College of Horticulture, Fujian Agriculture and Forestry University, Fuzhou, 350002 China

**Correction: aBIOTECH (2024) ** 10.1007/s42994-024-00146-8

The article “Characterization and functional analysis of gerbera plant defensin (PDF) genes reveal the role of GhPDF2.4 in defense against the root rot pathogen Phytophthora cryptogea”, written by Chunzhen Cheng, Huan Wu and Yongyan Zhang, was originally published electronically on the publisher’s internet portal on 31 March 2024 without open access. With the author(s)’ decision to opt for Open Choice the copyright of the article changed on 1 May 2024 to © The Author(s) 2024 and the article is forthwith distributed under a Creative Commons Attribution 4.0 International License, which permits use, sharing, adaptation, distribution and reproduction in any medium or format, as long as you give appropriate credit to the original author(s) and the source, provide a link to the Creative Commons licence, and indicate if changes were made.

The images or other third party material in this article are included in the article’s Creative Commons licence, unless indicated otherwise in a credit line to the material. If material is not included in the article’s Creative Commons licence and your intended use is not permitted by statutory regulation or exceeds the permitted use, you will need to obtain permission directly from the copyright holder.

To view a copy of this licence, visit http://creativecommons.org/licenses/by/4.0/.

