# Supplementary figures and images for: Characterization and functional analysis of gerbera plant defensin (PDF) genes reveal the role of GhPDF2.4 in defense against the root rot pathogen Phytophthora cryptogea
Source: aBIOTECH. 2024 Mar 31;5(3):325–38. doi: 10.1007/s42994-024-00146-8 (PMC11399501; doi:10.1007/s42994-024-00146-8)

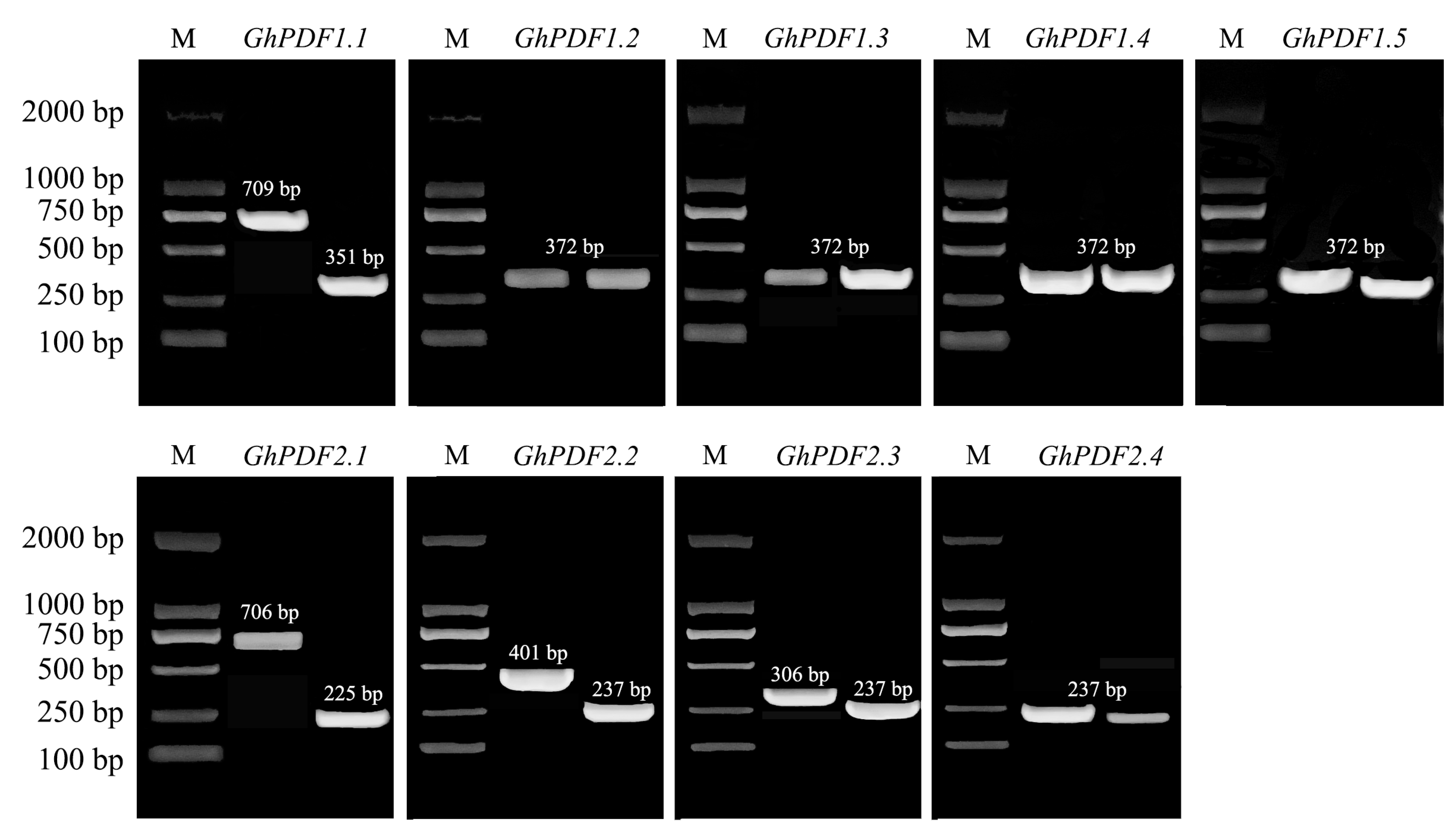

Supplement: Supplementary file 1 — Supplementary file1 (PNG 989 KB) [file 42994_2024_146_MOESM1_ESM.png]

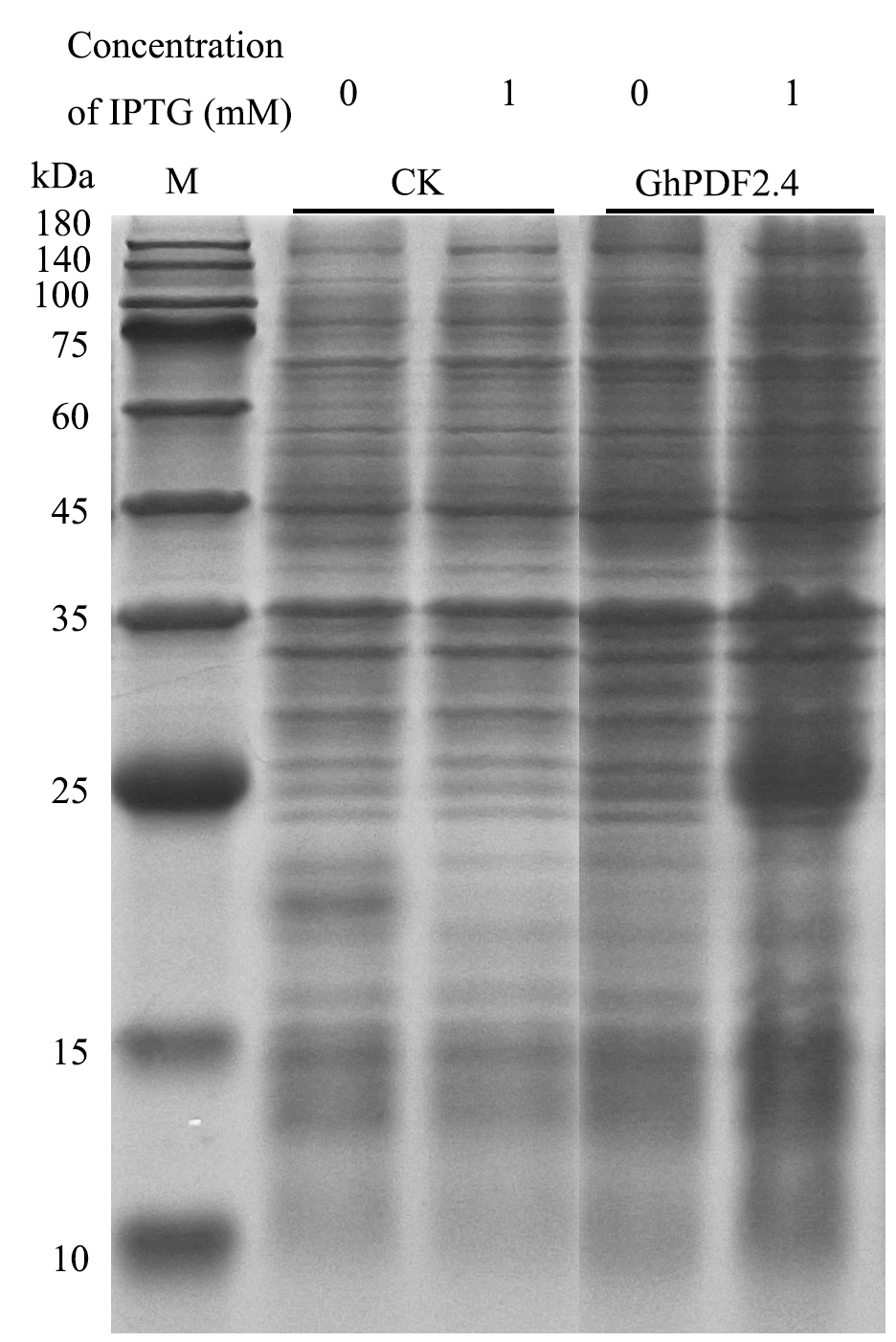

Supplement: Supplementary file 2 — Supplementary file2 (PNG 612 KB) [file 42994_2024_146_MOESM2_ESM.png]

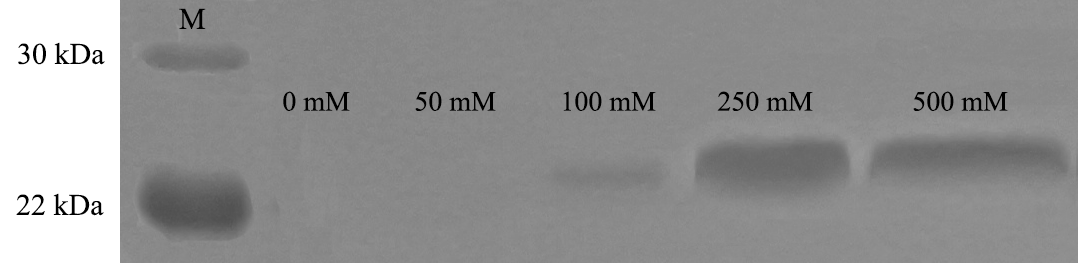

Supplement: Supplementary file 3 — Supplementary file3 (TIF 1660 KB) [file 42994_2024_146_MOESM3_ESM.tif]
