# Supplementary material for: Characterization and functional analysis of gerbera plant defensin (PDF) genes reveal the role of GhPDF2.4 in defense against the root rot pathogen Phytophthora cryptogea
Source: aBIOTECH. 2024 Mar 31;5(3):325–38. doi: 10.1007/s42994-024-00146-8 (PMC11399501; doi:10.1007/s42994-024-00146-8)
Supplement: Supplementary file 4 — Supplementary file4 (DOCX 35 KB) [file 42994_2024_146_MOESM4_ESM.docx]

**Table S1** Information for the primers used in this study.

| **Gene name** | **Primer Sequences (5’→3')** | **Target Length (bp)** | **Tm (℃)** | **Applications** |
| --- | --- | --- | --- | --- |
| *GhPDF1.1* | F: ATGGTGAAAAAATCGGTTGC | 351 | 60 | Gene cloning |
|  | R: TTAAGGATGGATTGGTCCGA |  |  |  |
| *GhPDF1.2* | F: ATGGTGAAAAAATCGGTTGC | 372 | 60 |  |
|  | R: TTAAGGTCCATGGGGATTGA |  |  |  |
| *GhPDF1.3* | F: ATGGTGAAGAAATCAGTTGC | 372 | 56 |  |
|  | R: TTAAGGTCCATGGGGATT |  |  |  |
| *GhPDF1.4* | F: ATGGTGAAGAAATCTATTG | 372 | 56 |  |
|  | R: CTAAGGTCCATGGGGATTG |  |  |  |
| *GhPDF1.5* | F: ATGGTGAAGAAATCAGTTGC | 339 | 56 |  |
|  | R: CTATGGCACT TTCTCGGCTT |  |  |  |
| *GhPDF2.1* | F: ATGGCAAGCAAGACCTTCG | 225 | 60 |  |
|  | R: TTAACAAAGCCTAGTGCAGAAAC |  |  |  |
| *GhPDF2.2* | F: ATGGCGAAGACGAGCTTCAAT | 252 | 56 |  |
|  | R: CTAACAGTTTTTGGTGCAAAAG |  |  |  |
| *GhPDF2.3* | F: ATGAGGCCTTCCATGAAGTTG | 237 | 60 |  |
|  | R: TTAACATTTTTTGGTGCAGAAAC |  |  |  |
| *GhPDF2.4* | F: ATGAGGCCTTCCATGAAGTT | 237 | 58.5 |  |
|  | R: TTAACATCTTTTGGTGCAGAAAC |  |  |  |
| *18S rRNA* | F: TCAAAGCAAGCCTACGCTCT | 125 | 60 | qRT-PCR in gerbera |
|  | R: GCTTTCGCAGTTGTTCGTCT |  |  |  |
| *GhPDF1.1* | F: GTTTGCGTTCCTTGTGCTCC | 146 | 60 |  |
|  | R: CCTCCCAAGCCTTGCATTGA |  |  |  |
| *GhPDF1.2* | F: TTCGTTTGCGTTCCTTG | 127 | 60 |  |
|  | R: GTCACAGTGTCGTGTGTTGC |  |  |  |
| *GhPDF1.3* | F: GACGTGGTCTGGAAATTGCG | 98 | 60 |  |
|  | R: TACCACTGCGCACATGACAA |  |  |  |
| *GhPDF1.4* | F: GACACTGCGACGATCAATGC | 130 | 60 |  |
|  | R: AGCTTGTCTTGGGCCAACTT |  |  |  |
| *GhPDF1.5* | F: TCATGTGCGTGGTGGTAAAC | 146 | 60 |  |
|  | R: GCACTTTCTCGGCTTTGACT |  |  |  |
| *GhPDF2.1* | F: GACCTTCGCTCTCTTCCTCAC | 80 | 60 |  |
|  | R: GGCACATTCTTGCTTCTGCTC |  |  |  |
| *GhPDF2.2* | F: CTGCTCCTCCTGGTTCCATA | 108 | 60 |  |
|  | R: CAGGCACTTCCCCTTGAAT |  |  |  |
| *GhPDF2.3* | F: GCGTTTCTTGTACTGATGTG | 141 | 60 |  |
|  | R: AGTCTTGCAAACACTACCAC |  |  |  |
| *GhPDF2.4* | F: ACAGAGATGGGTGGTGGAAT | 169 | 60 |  |
|  | R: TTTTGGTGCAGAAACAACGA |  |  |  |
| *NbEF-1α* | F: TGCTGCTGTAACAAGATGGATGC | 134 | 58 |  |
|  | R: GAGATGGGGACAAAGGGGATT |  |  | qRT-PCR in tobacco |
| *NbPR2* | F: TGATGCCCTTTTGGATTCTATG | 175 | 58 |  |
|  | R: AGTTCCTGCCCCGCTTT |  |  |  |
| *NbPR3* | F: CAGGAGGGTATTGCTTTGTTAGG | 222 | 58 |  |
|  | R: CGTGGGAAGATGGCTTGTTGTC |  |  |  |
| *NbACO* | F: GCTGAGGTTACTGATTTGGATTGG | 264 | 58 |  |
|  | R: TGGGCATGGTGGATAGTTGCT |  |  |  |
| *NbLOX1* | F: GTTGAAGGTTCTATCTGGCAGTTGG | 123 | 58 |  |
|  | R: TGTTGCGATCACGAATGGCTCTA |  |  |  |
| *GhPDF2.4* | F:GGCTGATATCGGATCCAGGACGTGCGAGTCACA | 141 | 57 | Prokaryotic expression |
|  | R:GTGCGGCCGCAAGCTTACATCTTTTGGTGCAGAA |  |  |  |
